# Real-time analysis and predictability of the health functional food market using big data

**DOI:** 10.1007/s10068-021-00999-5

**Published:** 2021-11-26

**Authors:** Sang-Soon Kim, Seokwon Lim, Sangoh Kim

**Affiliations:** 1 Department of Food Engineering, Dankook University, Dandae-ro 119, Dongnam-gu, Cheonan-si, Chungcheongnam-do 31116 Republic of Korea; 2 Department of Food Science and Biotechnology, Gachon University, Sungnamdero 1342, Sungnam-si, Gyeonggi-do 13120 Republic of Korea; 3 Department of Plant and Food Engineering, Sangmyung University, Sangmyeongdae-gil 31, Dongnam-gu, Cheonan-si, Chungcheongnam-do 31066 Republic of Korea

**Keywords:** Big data, Application programming interfaces, Online shopping, Health functional food, Programming

## Abstract

This study conducted a real-time analysis of the health functional food market using big data. To assess the scope of big data in market analysis, big data of the health food category were compared and analyzed with actual market data. Data were first collected using a program to obtain data, through application programming interfaces, followed by SPSS to compare and analyze the actual market index and shopping search word data. The correlation between the online search data and the actual market was high, indicating that online search data can be used to predict the trend of the actual market. Various types of data, such as items and major functional ingredients, can be collected and analyzed through the program developed for this study, which is also used to predict the market trend. The results demonstrate how APIs can be used to predict market size in the food industry effectively.

## Introduction

In March 2012, the Obama administration launched the Big Data Research and Development Initiative with a budget of 200 million USD (Jee and Kim, 2013). In Japan, big data development became an important axis of the national technology strategy in July 2012 (Oussous et al., 2018). In April 2012, the Korea Information Technology Agency (KISA) launched “Big Data Services.” In December 2013, the relevant government agencies jointly announced the Big Data Industry Development Strategy, and launched an initiative in June 2014 to use big data to innovate departmental tasks throughout the Ministry of Trade, Industry, and Energy (MOTIE) (Kim and Cho, 2017).

Globally, 2,500,000 terabytes of data are produced every day. Such an enormous amount of data can fill ten million Blu-ray discs; a stack of ten million Blu-rays would be equivalent to four times the height of the Eiffel Tower. Given the vastness of the data, it is likely to be considered as a population and not a sample. Using a workstation, such data can be processed quickly and provided for a specific purpose. Moreover, the cost of data production and consumption is low (Pandey et al., 2020). Big data find their application in various industrial aspects. Studies that used big data to determine the correlation between corporate-led and consumer-led activities crawled simple notification service (SNS) data, and found that activities, such as tagging, commenting, likes, and sharing SNSs affect corporate value. A study of the relationship that affects them revealed that SNS greatly contributes to future corporate performance (Park et al., 2016).

A beverage company that introduced a 2-L lemonade drink to the market in the shortest possible time and with limited research on how it can use big data to improve the new product development process, missed the potential benefits of big data (Jagtap and Duong, 2019). It is important to collect data of a high volume, variety, and velocity (known as 3 V) without invasion of privacy (Lu et al., 2014). Recently, some platforms such as NAVER (NV) provided application programming interfaces (APIs) to users (Lim and Park, 2011). The APIs provided by the organization portal gives access to big data at the population level. It can be used in various fields to derive interesting research results. One study aimed to determine the characteristics of urban commercial areas using online search results by age group (Lee and Lee, 2019). Another study established a system to quickly compare the second-hand transaction prices by developing a crawler system essential for the development of an integrated trading system for used goods through e-commerce (Park et al., 2020). Additionally, Verma et al. (2019) analyzed selected US stocks to predict daily gains in real time from Yahoo Finance using big data.

In the medical field, use of big data to prepare for risks by confirming the increase in the amount of internet searches related to the coronavirus disease 2019 (COVID-19) outbreak in Korea, has also been an interesting approach (Husnayain et al., 2020). Previously, Park et al. (2015) used an API to collect information related to agri-food to predict risk levels. Various types of data such as weather and digital tachographs, were collected from APIs and analyzed. Although various fields use big data analysis based on APIs, using it to access customers using portals in the food industry are scarce.

This study investigated the similarity between the actual market performance of health functional food (HFF) and the frequency of big data shopping searches using the search term frequency in the shopping API provided by NV, the top portal company in Korea. Further, market prediction was made using data science techniques after analyzing the similarity between the shopping search volume obtained with big data and the actual market result.

## Materials and methods

### Data resources

The size of the offline health functional food market in 2020 was sourced from the “2020 Health Functional Food Consumer & Market Research Report (HFFCMRR)” published by the Korea Health Supplements Association (Korea Health Supplements Association, 2021). The data comprised the purchase index of household health supplements surveyed using a specialized research institute and the production performance officially announced by the Korean Ministry of Food and Drug Safety (KMFDS) (Korea Health Supplements Association, 2021). Referring to HFFCMRR, the estimated HFF market size in 2020, based on survey data for the last five years, was KRW 4.99 trillion—a 6.6% increase from the previous year. The top-selling HFF items in 2020 were red ginseng, probiotics, vitamins (combined and single vitamins), and fats and oils containing eicosapentaenoic acid (EPA) and docosahexaenoic acid (DHA). Their combined market size was KRW 3211.7 billion, accounting for 64.5% of the market share.

This study used online shopping big data provided by NV. According to the survey conducted by Realmeter, a public opinion research organization in Korea, online shopping mall preferences were: Coupang at 19.7%, NV Shopping (NVS) at 15.8%, and Gmarket at 10.2%. Individuals aged under 50 years preferred Gyeongsangbuk-do/Gyeonggi/Incheon, students/office workers preferred Coupang, and those aged over 60 and housewives showed a higher preference for NVS. As of April 8, 2020, NVS secured KRW 12.5 trillion in payments, and accrued a record KRW 20.92 trillion in transactions in 2019. NVS has now emerged as the top e-commerce company in Korea. Hence, NVS search data can be considered vast and reliable. Through NVS data, it would be possible to predict consumer shopping trends accurately, easily, and quickly, not only in HFF but also in other food categories.

### Data collection program development

It is necessary to obtain authority for the OPEN API to use big data provided by NV. After following due procedures, data were extracted through a program created using Python3 and QT GUI. The program enabled easy access and storage of the accumulated data. NV restricts free users to four product items that can be called through the API, and 1,000 calls per day. Additionally, the returned value is a relative ratio based on the highest 100% of the retrieved data; the absolute value cannot be known. In other words, because each item has a relative value, it was impossible to compare the absolute values with other items. After analyzing returned information, this problem was solved by setting the search ratio as a standard for the Maximum Fixed Item's search ratio, making it possible to determine the absolute value for a comparison between other items. Thus, to achieve absolute comparison, the program applied this concept by implementing the following algorithm:

$$Ratio_{absolute} = \frac{{Ratio_{relative} }}{{Ratio_{fixed,max} }}.$$

**Fig. 1:**
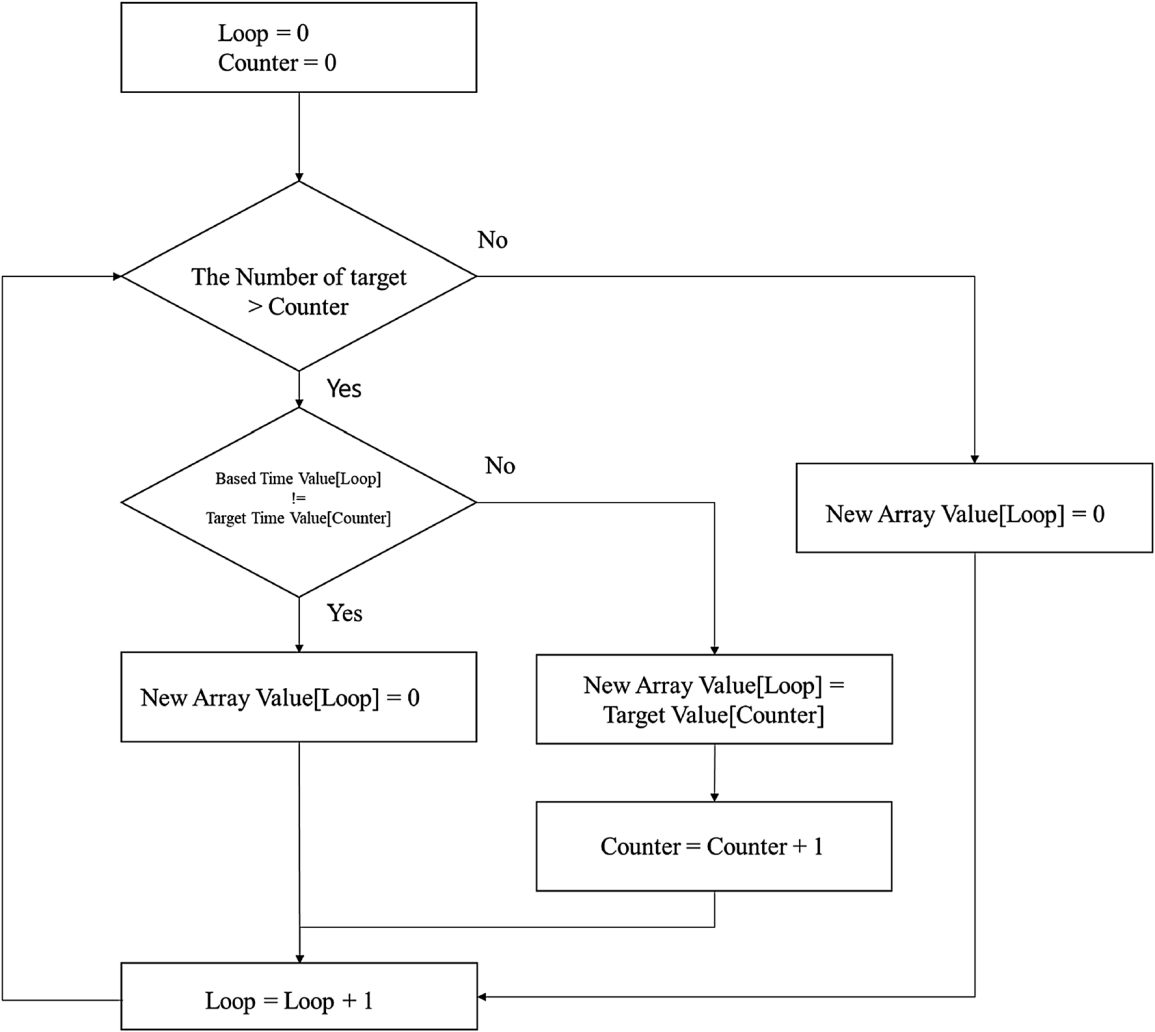
Calibration algorithm to fit the size of the array

However, the issue with target item's search ratio obtained through API was that when there was no search volume or no data in a new category, the result showed an empty space, rather than “0(zero).” When merging them, a data fall error occurred due to the mismatch in array size. To prevent this, the dimensions of all arrays was corrected through an algorithm, as shown in Fig. 1. The missing part of the array was matched based on the size of the returned array of the Fixed Item.

### Data analysis

The final data obtained after correction were stored as comma-separated values. The data can be analyzed using statistical programs, such as SAS, SPSS, or R.

**Fig. 2:**
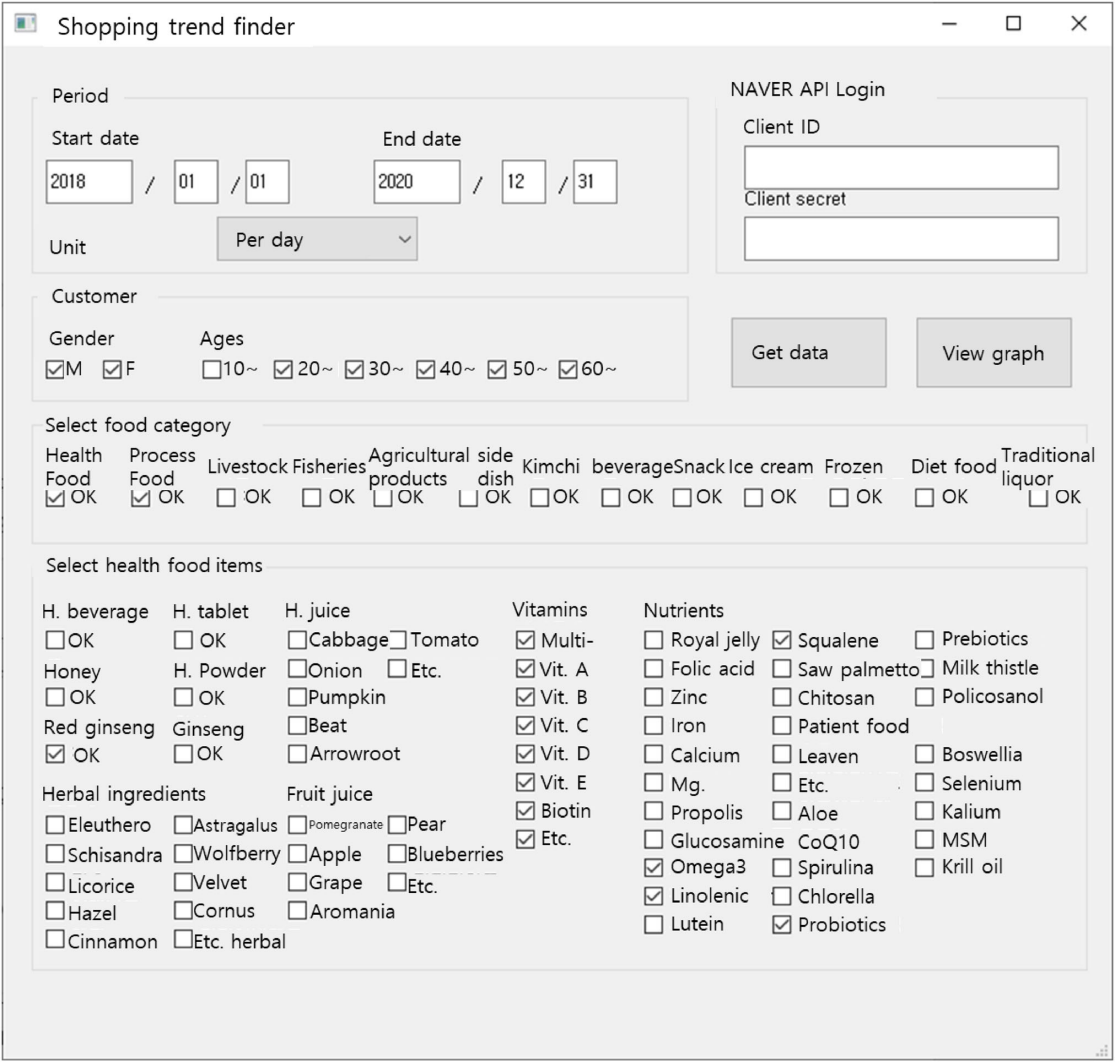
Program created using Python3 and QT GUI to collect NAVER shopping big data

Using a program with a GUI (as shown in Fig. 2), 8,760 secondary-processed search rate data values were collected and analyzed under the following conditions.

- Collection period: 2018–2020.
- Data unit: Daily.
- Target: Men and women of age ≥ 20 years.
- Items: HFF, Process Food (PF), Red Ginseng, Vitamins, Omega 3, Gamma linolenic Acid, Squalene, Probiotics.

Using IBM's SPSS Statistics (24, IBM Corp., Armonk, NY, USA), the results of the online shopping search rate were plotted in a boxplot and chart graph, and the average and Pearson correlation were evaluated (*p* < 0.05).

## Results and discussion

### The correlation between online search and actual market (total HFF market)

**Fig. 3:**
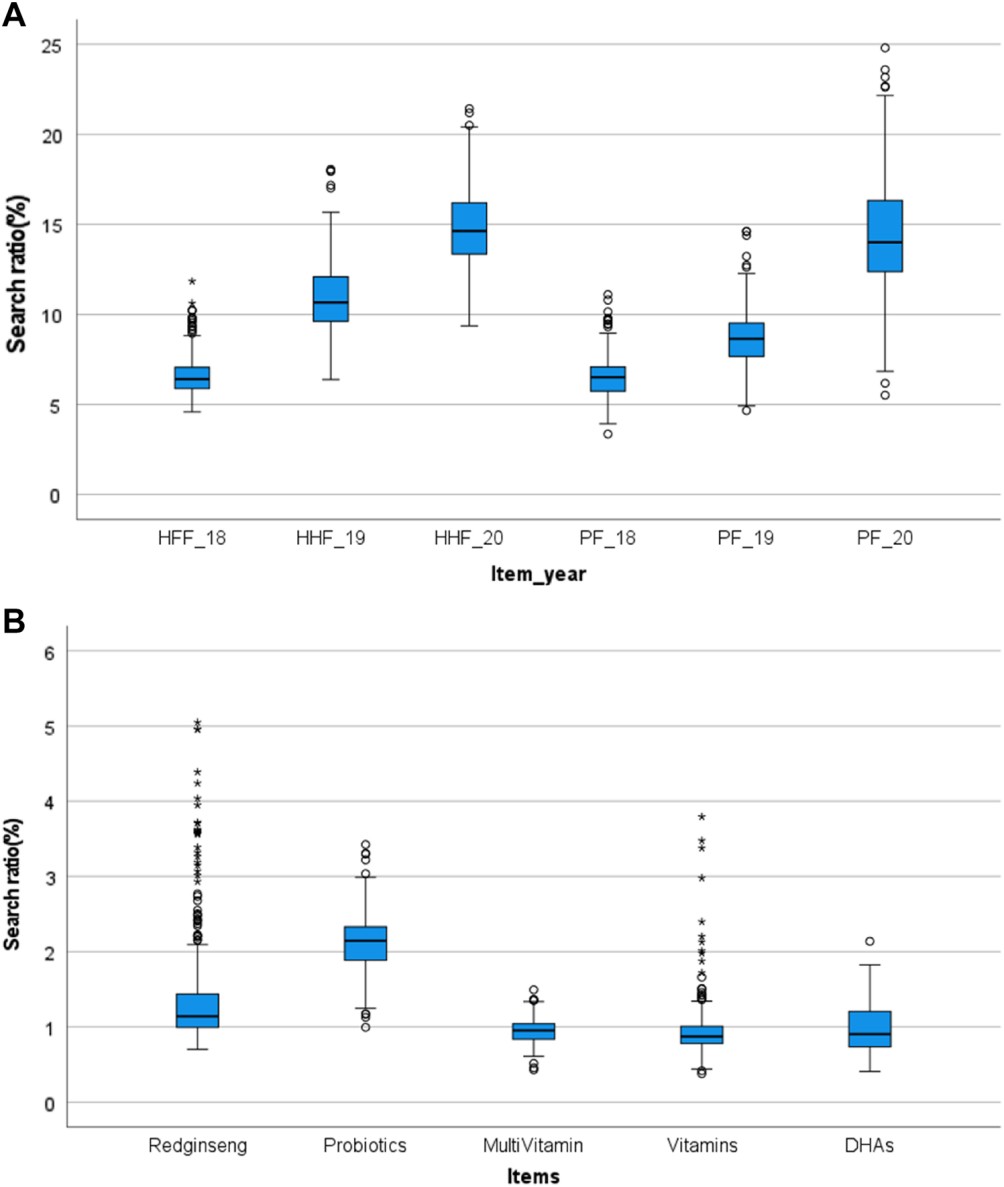
The search rate of health functional food (HFF) and processed food (PF) by year (A) (n = 365) and the HFF by functionality in 2020 (B). The asterisks (*) means the significant difference (*p* < 0.05)

The three-year health food category data obtained through the NV API were separated by year and displayed using a boxplot (Fig. 3A). From a peripheral view, the online search volumes of HFF and PF exhibited a steady increase. However, observing the changes in these mean values revealed variations of 6.58–14.78% and 6.51–14.43% for HFF and PF, respectively. Analyzing the Pearson correlation for the actual market (HFFCMRR) and data of the online search volume (NVS) using SPSS confirmed that the correlation between online search and the actual market for the entire HFF category was high (0.990 of the Pearson correlation coefficient). This result indicated that the online search rate can be used to predict the trend of the actual market. Though the search rate of PF has steadily risen, HFF search volume increased rapidly because COVID-19 has driven purchases of HFF. Given the changes in consumption patterns, re-analyzing the big data of credit card usage was it was inevitable to (Jo et al., 2021).

### The correlation between online search and the actual market (by function)

**Fig. 4:**
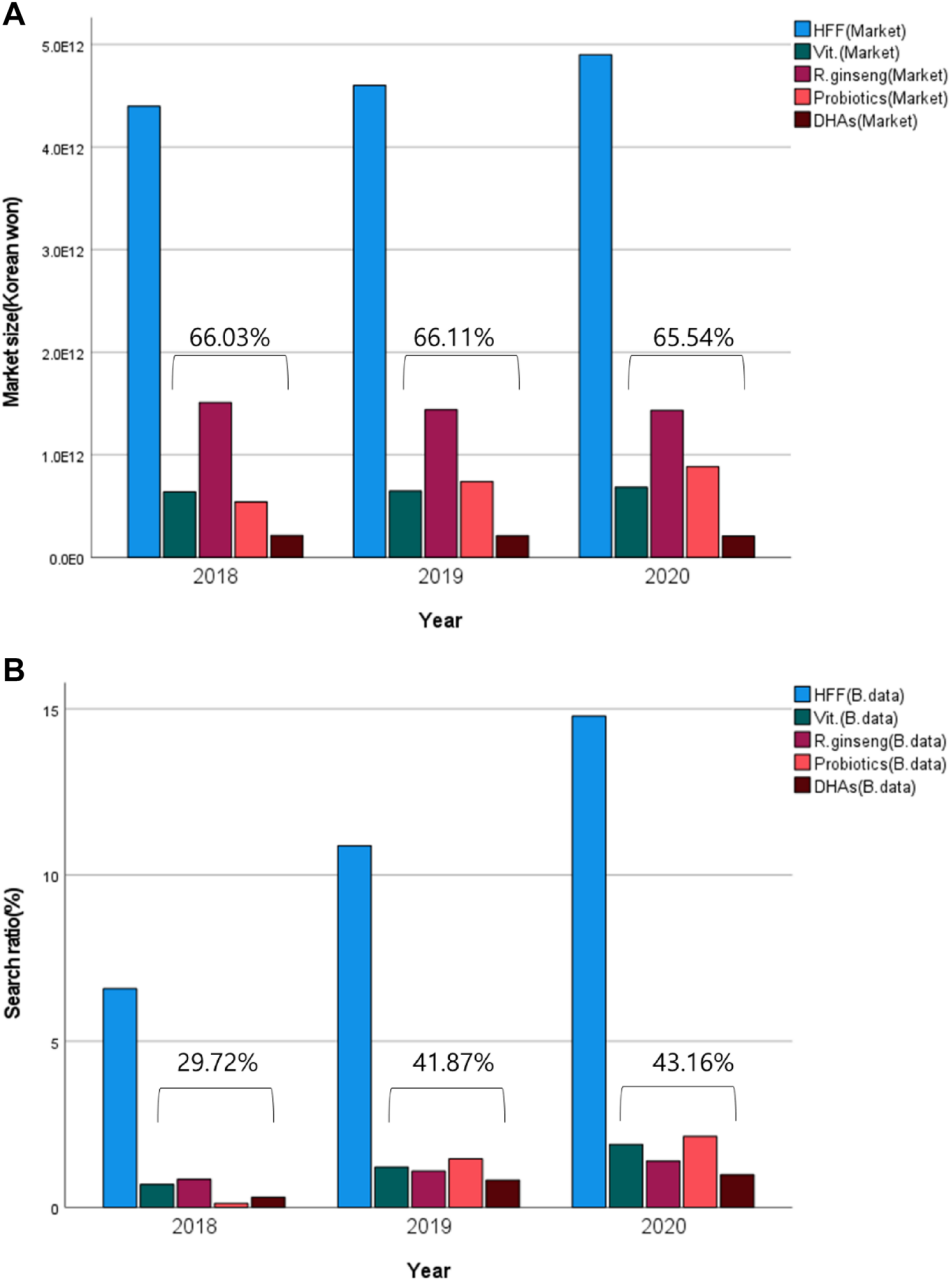
Changes by year in actual market (A) and big data search volume (B) of health functional food (HFF). The sum of ratios of four major health foods in total health food each year were indicated as numbers

Figure 4 illustrates the data on the actual market (HFFCMRR) and the search rate (NVS), calculated with big data and organized by year schematically. Functional substances used in foods, such as red ginseng, probiotics, vitamins, and DHA, as expressed in the HFFCMRR data, were analyzed and compared with NVS data.

In the big data search rate, red ginseng changed from 0.84% to 1.39%, probiotics from 0.12% to 2.13%, vitamins from 0.69% to 1.89%, and DHAs from 0.31% to 0.97%. The change was then compared to the actual market change, which was KRW 1.51 trillion to KRW 1.43 trillion for red ginseng, KRW 0.54 trillion to KRW 0.89 trillion for probiotics, KRW 0.64 trillion to KRW 0.68 trillion for vitamins, and KRW 0.21 trillion to KRW 0.20 trillion for DHAs. The Pearson correlation coefficients between the actual market and the big data for functional foods were -0.874 for red ginseng, 0.996 for probiotics, 0.964 for vitamins, and -0.933 for DHAs. Additionally, the ratio of the four major health foods to the total HFF accounted for about 66% in the actual market data; however, in the big data, there are many differences, such as 29.72% in 2018, 41.87% in 2019, and 43.16% in 2020.

The Pearson correlation coefficients between the actual market and the big data for functional foods were -0.874 for red ginseng, 0.996 for probiotics, 0.964 for vitamins, and -0.933 for DHAs. In the second result, the results differed in the share of HFF based on the total HFF. These results highlighted an issue with the correlation analysis result.

There could be four possible reasons. First, the HFFCMRR data were calculated both online and offline, whereas the big data was collected solely from online data search of NVS. Second, red ginseng had a market price 2–3 times higher than that of probiotics. This is because the actual market size was calculated based on the sales price, whereas online search volume accounted for searches by word, regardless of the actual price. Third, the shopping search data and category classification of NV is still in progress. When data were obtained through the actual API, only the data since 2018 responded normally in all areas. For the 2018 data, the search volume was displayed as “0” due to the late category classification. Finally, the HFF sold in the HFF category included not only KFDA-certified foods, but also foods that contain non-licensed functional ingredients sold in a significant amount.

The establishment of increasingly sophisticated and refined databases over time could potentially address these problems. With this evidence, the share of major health supplements, compared to HFF in the NVS data, were 29.72%, 41.87%, and 43.16% in 2018, 2019, and 2020, respectively, closing the gap with the 66% actual share in the HFFCMRR data. In other words, there was visible progress in data enhancement.

In the correlation analysis using HFF functionality, vitamins and probiotics exhibited a high positive correlation, whereas red ginseng and DHAs showed a negative correlation. Similar to the previous situation, it seemed that the trend would increase consistently over time. Further, when analyzing the trend in the HFFCMRR data, red ginseng deceased from KRW 1.50 trillion in 2018 to KRW 1.43 trillion in 2020, compared to the increase in the amount of red ginseng searches in online search volume from 0.83% in 2018 to 1.38% in 2020. In terms of probiotics, the HFFCMRR data increased from KRW 0.54 trillion in 2018 to KRW 0.88 trillion in 2020. The online search volume for red ginseng also displayed an overwhelming increase from 0.12% in 2018 to 2.13% in 2020. The significant increase in the degree of interest has been evident in Korea.

### Trend analysis and search rate of major functional ingredients in 2020

**Fig. 5:**
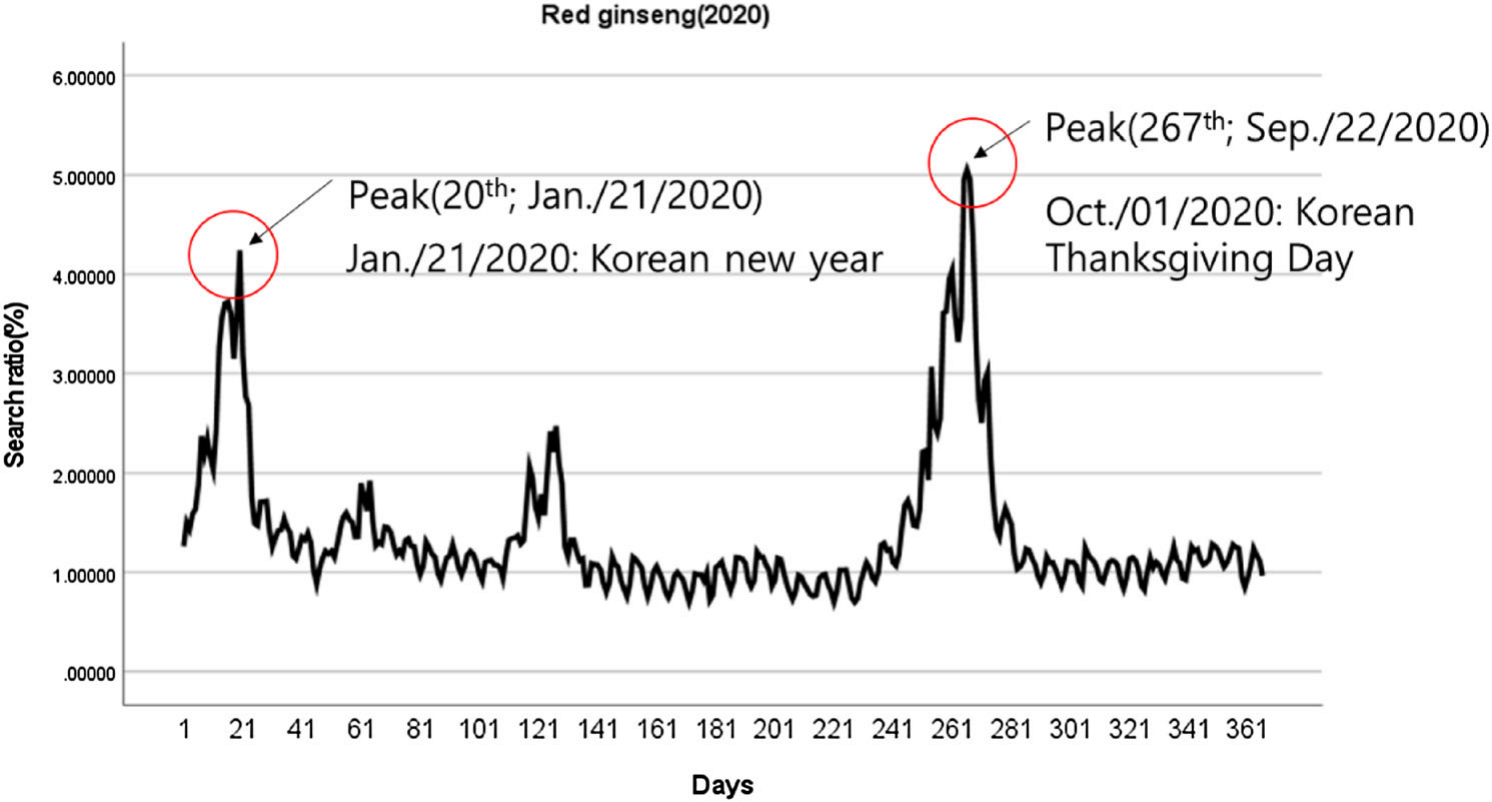
The time series graph of red ginseng search rate in 2020

Figure 3B provides a boxplot of the dietary supplement search ratios from January to December 2020. Red ginseng was 1.38%, probiotics was 2.13%, multivitamin was 0.95%, vitamins were 0.94%, and DHAs was 0.97%. The outlier values in the boxplot can be explained by referring to the time series graph in Fig. 5. A close look at the graph reveals a large search volume at a specific time. As the search volume exploded on the 20th and 267th days of 2020, many outliers occurred, which coincides with the Korean New Year and Korean Thanksgiving Day, the major Korean holidays. Since the search volume does not reflect the volume of purchases, red ginseng, traditionally the top-selling item in HFF, is far behind probiotics in terms of average value; however, it can be calculated by reflecting the Upper Specification Limit (USL), considering the deviation. The conversion results were 2.12% for red ginseng, 2.49% for probiotics, 1.10% for multivitamins, 1.29% for vitamins, and 1.28% for DHAs.

In conclusion, the results indicated that the real-time prediction of the HFF market is possible through big data provided by portal sites. However, the results confirmed that category enhancements and product classifications remain insufficient. Sakar et al. (2019) suggested a method to predict online shoppers' purchase intentions in real time using big data and artificial intelligence based on deep learning. Although this study employed NV's shopping search API, it appears to be insufficient as a research method due to lack of historical data. Nevertheless, with the establishment of reliable databases, these problems should diminish in the future. Interesting research results can then be obtained at regular intervals. From the results of the present study, other domestic and foreign portals are likely to introduce APIs, and research on various topics is expected to be active in the food industry in the future.
